# Prognosis of non-small cell lung cancer patients with bone oligometastases treated concurrently with thoracic three-dimensional radiotherapy and chemotherapy

**DOI:** 10.1186/1748-717X-9-147

**Published:** 2014-06-24

**Authors:** Wei-Wei Ouyang, Sheng-Fa Su, Zhu Ma, Yin-Xiang Hu, Bing Lu, Qing-Song Li, Yi-Chao Geng, Hui-Qin Li

**Affiliations:** 1 Department of Thoracic Oncology, Affiliated Hospital of Guiyang Medical College, and Guizhou Cancer Hospital, 1 Beijing Road West, Guizhou Guiyang, China

**Keywords:** Non-small Cell Lung Cancer, Bone Metastases, Thoracic Radiotherapy, Chemotherapy

## Abstract

**Background:**

To evaluate the efficacy of three-dimensional radiotherapy for non-small cell lung cancer (NSCLC) patients with bone metastases.

**Methods:**

Clinical data for 95 NSCLC patients with bone metastases were collected and prognostic factors were analyzed. All patients received radiation to their thoracic primary tumor and ≥2 cycles of chemotherapy.

**Results:**

Of these 95 patients, 47 patients had only bone metastases and 48 had both bone metastases and other organ metastases. Univariate analysis showed that factors that statistically significantly contributed to patients having longer overall survival (OS) included receiving a radiation dose to the primary tumor ≥63 Gy, responding to treatment and receiving ≥4 cycles of chemotherapy (*p* = 0.001, *p* = 0.037 and *p* = 0.009, respectively). A radiation dose to the primary tumor ≥63 Gy remained significant for patients with bone metastases only as well as those with bone and other organ metastases when they were analyzed separately (*p* = 0.045 and *p* = 0.012, respectively). For patients with bone metastases only, those with T1-2 tumors had longer OS than those with T3-4 (*p* = 0.048); and patients who received ≥4 cycles chemotherapy compared with those who received <4 cycles had similar OS (*p* = 0.385). On multivariate analysis, only a radiation dose ≥63 Gy (*p* = 0.028) and having only bone metastases (*p* = 0.006) were independent prognostic factors for better OS.

**Conclusions:**

A radiation dose to the primary tumor ≥63 Gy and having only bone metastases were associated with better OS in NSCLC patients with bone metastases. For patients with bone metastases only, besides radiation dose, T status was also correlated with OS, whereas the number of chemotherapy cycles was not. Therefore, aggressive thoracic radiation may play an important role in improving OS.

## Introduction

Approximately 55% of patients newly diagnosed with non-small cell lung cancer (NSCLC) have distant metastases [1]. System chemotherapy is the main treatment modality for stage IV NSCLC. The response rate to platinum-based doublet chemotherapy for stage IV NSCLC is approximately 30–40%, and this treatment produces a median survival time (MST) of 8–10 months [2,3]. Moreover, the survival duration has not obviously increased with chemotherapy treatment for stage IV NSCLC patients over the past 10–15 years [4]. Different third-generation chemotherapy regimens have similar efficacy, indicating that the efficacy of a chemotherapeutic approach has reached a plateau. However, the metastatic status of NSCLC patients shows variability, and how to treat stage IV NSCLC patients with radiation therapy is not well defined.

We investigated clinical metastases features in 546 patients with stage IV NSCLC and 53.8% (294/546) of patients had bone metastases, bone being the most common metastatic site [5]. Patients with lung cancer who develop bone metastases have a poor prognosis; the MST ranges from 7.0 to 8.0 months [6,7]. Recent publications also have reported that radiation of the primary tumor may prolong survival time in certain patients with stage IV NSCLC [8-10]. In this context, we performed this study to investigate outcomes and prognostic factors for NSCLC patients with bone oligometastases at diagnosis, who received radiation therapy for their thoracic primary tumor.

## Methods

### Patient selection and pretreatment evaluation

Ninety-five patients who came to the hospital from January 2003 to July 2010 with stage IV NSCLC and who fulfilled all of the following criteria were included in this study. (1) Pathologically or cytologically confirmed diagnosis of NSCLC; (2) newly diagnosed stage IV disease according to the staging system of the 2002 American Joint Committee on Cancer; (3) aged between 18–80 years; (4) Karnofsky Performance Status (KPS) score ≥70%, as well as a weight loss of no more than 10% during the 6 months prior to therapy; (5) bone metastases at ≤5 sites; (6) adequate bone marrow, liver and renal function; (7) no radiotherapy or chemotherapy contraindications; (8) thoracic radiotherapy using either three-dimensional conformal radiation therapy (3D-CRT) or intensity-modulated radiation therapy (IMRT); and (9) treatment with at least two cycles of chemotherapy. Exclusion criteria were as follows: (1) history of a thoracic operation, radiotherapy or chemotherapy; (2) pregnant or lactating; and (3) previous malignancy or other concomitant malignant disease. The Institutional Review Board of the Affiliated Hospital of Guiyang Medical College and Guizhou Cancer Hospital China approved this study, and the informed consent was obtained from all patients.

Pretreatment evaluation included a complete physical examination and hematologic and biochemistry profiles. Fiberoptic bronchoscopy examination and contrast-enhanced computed tomography (CT) of chest were performed to accurately evaluate the extent of the primary tumor and regional lymph nodes. Bone scintigraphy, contrast-enhanced CT of the abdominal region and magnetic resonance imaging (MRI) of the brain were routinely used to detect distant metastases. If a PET/CT scan was done, then bone scintigraphy and contrast-enhanced CT scans of the abdominal region were not necessary. Additional investigations were performed if indicated. Positive PET/CT or bone scan findings for bone metastases also required other additional radiologic confirmation (e.g., MRI of bone). Clinical characteristics of the 95 patients are detailed in Table 1.

**Table 1:** Clinical characteristics of 95 NSCLC patients with bone metastases

**Characteristic**	**Whole group**	**Only bone metastases**		**Bone with other organ metastases**
		**Metastases to 1–2 sites**	**Metastases to ≥2sites**	
Gender				
Male	68	18	18	32
Female	27	2	9	16
Age (years)				
Range (median)	30 ~ 78(59)	40 ~ 78(62)	41 ~ 72(59)	30 ~ 76(58)
<60	50	9	14	27
≥60	45	11	13	21
Pathological type				
Squamous	31	7	10	14
Non-squamous	64	13	17	34
T stage				
T_1–2_	36	11	10	15
T_3–4_	59	9	17	33
N stage				
N_0–1_	14	3	4	7
N_2–3_	81	17	23	41
GTV	95	17 ~ 489(142)	28 ~ 604(147)	28 ~ 628(179)
KPS				
70	39	5	9	25
>70	56	15	18	23
Prescribed dose				
Range (median)	9 ~ 76(63)	23 ~ 76(63)	9 ~ 72(63)	9 ~ 68(58)
<63 Gy	47	10	8	29
≥63 Gy	48	10	19	19
Treatment response of primary tumor				
CR + PR	66	15	18	33
SD + PD	29	5	9	15
Chemotherapy				
Range (median)	2 ~ 5(4)	2 ~ 4(3)	2 ~ 4(4)	2 ~ 5(4)
2-3 cycles	43	10	9	24
≥4 cycles	52	10	18	24
Radiation to metastases				
Yes	15	9	3	3
No	80	11	24	45

Note: GTV from a minimum ~ maximal value cm^3^ (median).

### Radiotherapy protocol

All patients were immobilized in the supine position with a T bar, wing board and Vac-lock cradle. Images with contrast were obtained from the CT simulator for treatment planning purpose. All patients were scanned with serial 5-mm slices from the hyoid bone through the third lumbar vertebra. All patient 3D-CRT or IMRT treatment plans were performed using the ADAC pinnacle^3^ planning system (version 7.4 f) and dose distribution was computed with tissue heterogeneity correction. The gross tumor volume (GTV) included thoracic primary tumors and hilar or mediastinal lymph nodes with a short-axis diameter of at least 1 cm on CT, and the planning target volume (PTV) was defined as the GTV plus a 1.5-cm margin for setup uncertainty and respiratory motion. Radiation was delivered with a linear accelerator using 6 MV photons. V20 (percentage of the total lung volume receiving ≥20 Gy), the maximal point dose of spinal cord and mean esophagus dose were required to be ≤32%, 50 Gy and ≤35 Gy, respectively, for the individual treatment plan. The prescribed dose encompassed at least 95% of PTV. Thoracic radiation was delivered in 2 Gy daily fractions (5 days each week) and patients received thoracic radiation of at least a dose of 40 Gy in 20 fractions. Thoracic radiation treatment was implemented concurrently with chemotherapy. The fractionated radiotherapy dose for metastatic tumors ranged from 3 to 10 Gy/fraction with 1 fraction/day, and the total prescribed radiotherapy dose for metastatic lesions ranged from 20 to 60 Gy. Radiation to metastatic lesions was implemented concurrently or sequentially with chemotherapy.

### Chemotherapy protocol

All patients received platinum-based doublet chemotherapy and the selection of regimens was according to prior studies [3,11]. The commonly used regimens and usage were as follows: 135–175 mg of paclitaxel (P) per square meter of body surface area (mg/m^2^) or 75 mg/m^2^ of docetaxel (D) administered on day 1, followed by 80 mg/m^2^ of cisplatinum (C) or carboplatin (Cb) at a dose of 300–350 mg/m^2^ administrated on day 2, and vinorelbine (V) at a dose of 25 mg/m^2^, administered on days 1 and 8 during thoracic radiotherapy given every 21–28 days. Concurrent thoracic radiation was given within 1 week following the start of chemotherapy. After completion of thoracic radiotherapy, patients demonstrating a response or stable disease continued on chemotherapy for up to 4–6 cycles, whereas patients who experienced progressive disease or unacceptable toxicity were transferred to second-line therapy. Platinum and taxane-based chemotherapy were the main regimens used in the current study. PC or PCb regimens were used in 38 cases, DC or DCb regimens in 51 cases and the VC regimen in six cases. In total, 45% of patients received two or three cycles of chemotherapy, and 55% of patients received four or five cycles of chemotherapy. The total number of cycles was 315 (mean per patient, 3.3).

### Statistical analysis

The Statistical Package for Social Sciences, version 13.0 (SPSS, Chicago, IL) was used for statistical analysis. The Kaplan-Meier method was used to calculate overall survival (OS) and compared using the log-rank test. Factors with *p* <0.1 were included in multivariate analysis. The Cox model was used for multivariate analysis of OS. All statistical tests were two-sided, and *p* <0.05 was considered statistically significant.

## Results

The last follow-up was in November 2012. The follow-up periods ranged from 2.0 to 76.0 (median, 11.0) months. At the time of the last follow-up, 92 patients had died, one patient was lost to follow-up at 20 months after finishing treatment (who was in the group of bone and other organ metastases) and two patients were still alive with survival times of 47 and 76 months. For all patients, the MST was 11.0 months (95% confidence interval (CI), 8.5–13.5) and the 1-, 2-, and 3-year OS rates were 43.6, 16.8 and 8.5%, respectively. The 1-, 2- and 3-year OS rates were 58.1, 24.8 and 15.8%, respectively for patients with bone metastases only and the MST was 14 months (95% CI, 10.3–17.7). For patients who had bone and other organ metastases, the 1-, 2- and 3-year OS rates were 31.8, 9.8 and 0.0%, respectively and the MST was 8 months (95% CI, 5.6–10.4) (*χ*^*2*^ = 10.092, *p* = 0.001). For patients with bone metastases only, the OS for patients with metastases in 1–2 sites was similar to those with metastases in ≥3 sites (*χ*^*2*^ = 0.029, *p* = 0.866). The median GTV was 159 cm^3^ (17–628 cm^3^). The 1-, 2-, and 3-year OS rates for patients with GTV <159 cm^3^ compared with those with GTV ≥159 cm^3^ were 54.8 versus 37.5%, 19.4 versus 9.4% and 11.9 versus 3.1%, respectively, and the MST was 14 months (95% CI, 9.6–18.4) versus 9 months (95% CI, 6.7–11.3), respectively (*χ*^*2*^ = 3.281, *p* = 0.070). The status of the primary tumor and metastatic lesions could be evaluated in 58 of the 92 patients who had died. Of these, six cases had progressive disease of the primary tumor without developing any new metastases, and of these six cases, four involved a radiation dose <40 Gy, seven cases had progressive disease of the primary tumor and initial metastatic lesions, six cases had progressive disease of the primary tumor and new metastases, and 39 cases had new metastases in initially involved or uninvolved organs.

The 1-, 2-, and 3-year OS rates for patients who received a radiation dose ≥63 Gy to the primary tumor compared with those who received a radiation dose <63 Gy were 60.2 versus 26.2%, 21.8 versus 11.9% and 12.5 versus 4.0%, respectively and the MST was 15 months (95% CI, 11.9–18.1) versus 9 months (95% CI, 6.6–11.4), respectively (*χ*^*2*^ = 11.038, *p* = 0.001). There was a significant association between treatment response of the primary tumor and OS; patients who responded (complete remission + partial remission) had longer OS than those without response (stable disease + progressive disease) (*χ*^*2*^ = 4.364, *p* = 0.037). Radiation to metastatic sites was not significantly correlated with OS in patients who had both bone and other organ metastases (*χ*^*2*^ = 0.259, *p* = 0.611). However, for patients with bone metastases only and who received radiation to metastatic sites, there was a trend towards a better OS (*χ*^*2*^ = 2.757, *p* = 0.097).

**Figure 1:**
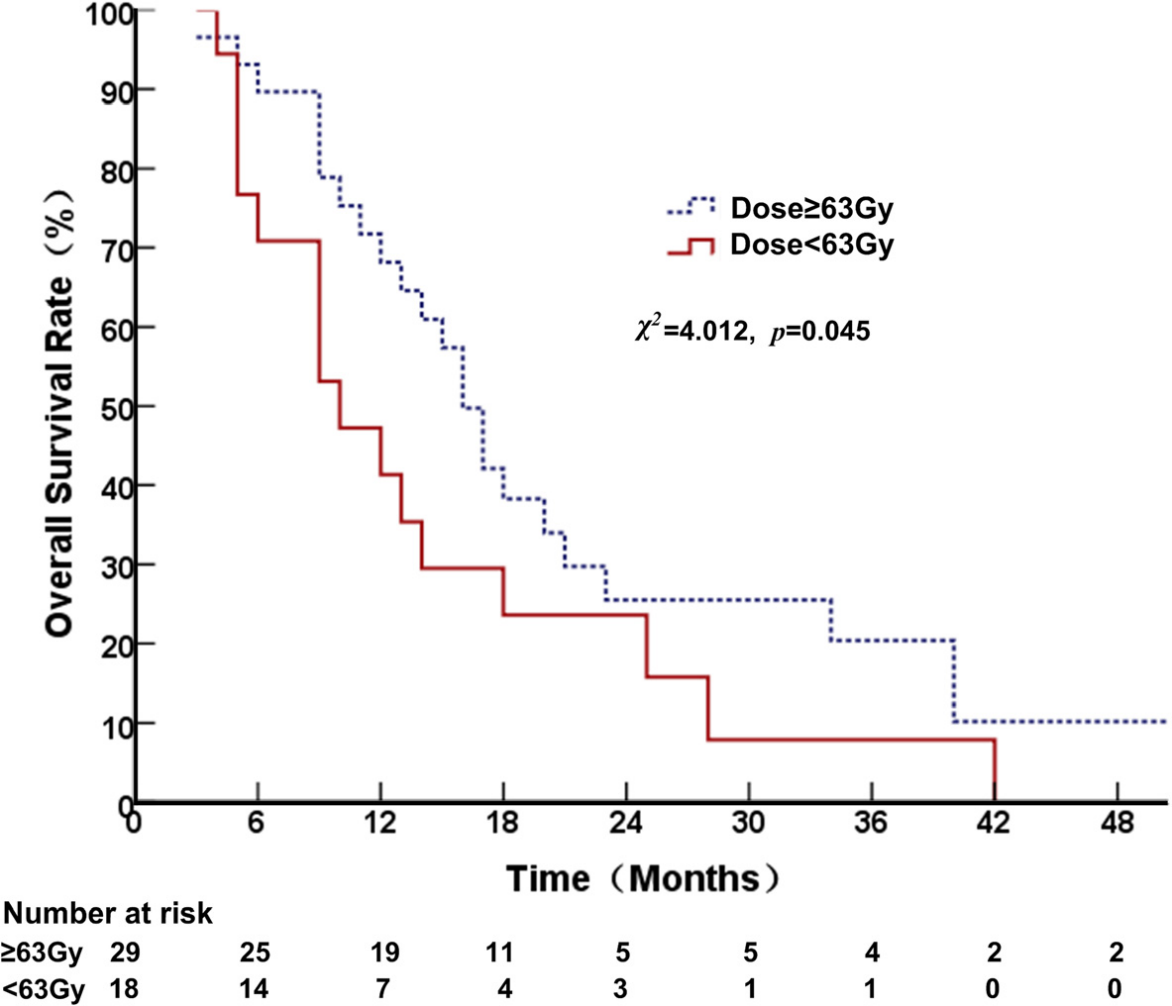
Comparison of overall survival curves at different radiation doses in patients with only bone metastases.

**Figure 2:**
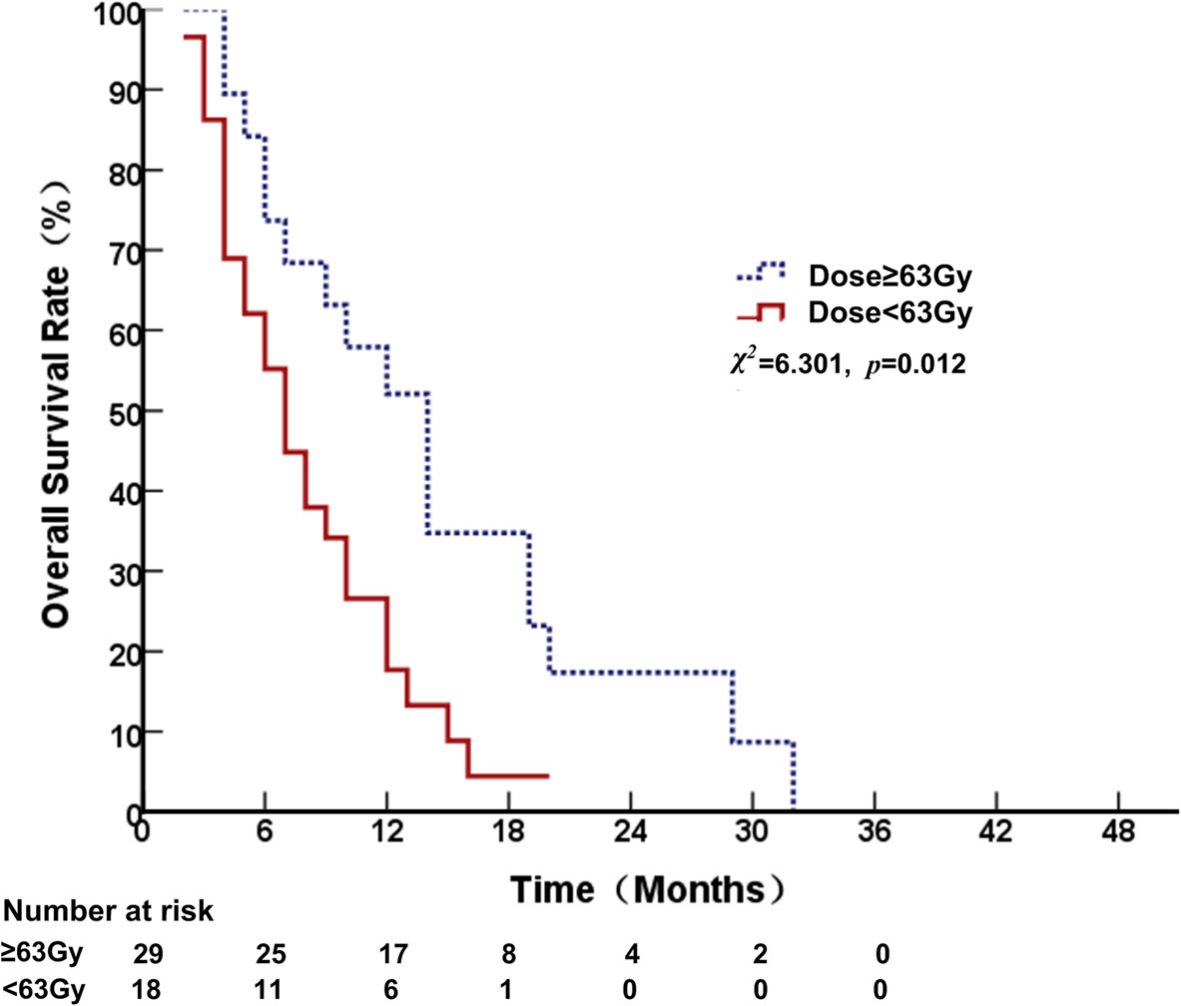
Comparison of overall survival curves at different radiation doses in patients with metastases to both bone and other organs.

A radiation dose to primary tumor ≥63 Gy remained significant for OS when patients with only bone metastases and those who had bone and other organ metastases were analyzed separately. For patients with bone metastases only, the 1-, 2- and 3-year OS rates were 68.1, 25.5 and 20.4%, respectively and the MST was 16 months (95% CI, 14.1–17.9) for those who received radiation ≥63 Gy, and the 1-, 2- and 3-year OS rates were 41.3, 23.6 and 7.9%, respectively for those who received radiation <63 Gy and the MST was 10 months (95% CI, 4.8-15.2) (*χ*^*2*^ = 4.012, *p* = 0.045, Figure 1). For patients who had bone both and other organ metastases, the 1-, 2- and 3-year OS rates were 52.1, 17.4 and 0.0%, respectively, and the MST was 14 months (95% CI, 9.6–18.4) for those who received radiation ≥63 Gy, whereas it was 26.6, 0.0 and 0.0%, respectively for those who received <63 Gy and the MST was 7 months (95% CI, 4.8–9.2) (*χ*^*2*^ = 6.301, *p* = 0.012, Figure 2).

The 1-, 2- and 3-year OS rates for T1-2 patients compared with T3-4 were 50.0 versus 39.5%, 27.8 versus 9.3% and 18.5 versus 2.3%, respectively and the MST was 14 months (95% CI, 9.6–18.4) versus 9 months (95% CI, 6.1–11.9), respectively (*χ*^*2*^ = 3.912, *p* = 0.048). For patients with bone metastases only, patients with T1-2 tumors had longer OS than those with T3-4, and the 1-, 2- and 3-year OS rates were 66.7 versus 46.2%, 38.1 versus 10.8% and 25.4 versus 5.4%, respectively, and the MST was 17 months (95% CI, 12.4–21.6) versus 11 months (95% CI, 7.1–14.9), respectively (*χ*^*2*^ = 3.904, *p* = 0.048). For patients who had both bone and other organ metastases, T-classification of the primary tumor was not correlated with OS (*χ*^*2*^ = 0.001, *p* = 0.962). Patients with a KPS score >70 showed borderline significantly better OS than those with a KPS equal to 70 (*χ*^*2*^ = 2.955, *p* = 0.086).

OS was significantly prolonged in patients who received ≥4 cycles chemotherapy, and the 1-, 2- and 3-year OS rates were 57.1, 20.3 and 11.6%, respectively, whereas the OS rates were 27.2, 12.4 and 4.6% for those who received <4 cycles, respectively, and the MST was 14 months (95% CI, 11.6–16.4) versus 8 months (95% CI, 4.9–11.1) (*χ*^*2*^ = 6.800, *p* = 0.009), respectively. Similarly, among patients who had both bone and other organ metastases, OS was significantly prolonged in those who had received ≥4 cycles of chemotherapy versus those who had received <4 cycles, and the MST was 14 months (95% CI, 8.7-19.3) versus 6 months, respectively (95% CI, 3.1–8.9) (*χ*^*2*^ = 9.706, *p* = 0.002). However, this difference was not statistically significant in patients with bone metastases only, and the MST was 14 months (95% CI, 10.3–17.7) versus 12 months (95% CI, 6.8–17.2) (*χ*^*2*^ = 0.756, *p* = 0.385). Univariate analysis revealed that sex, age, pathology type and N stage were not associated with OS. Multivariate analysis revealed that a radiation dose ≥63 Gy (*p* = 0.028) and bone metastases only (*p* = 0.006) were independent prognostic factors for better OS, and GTV (*p* = 0.056) and treatment response of the primary tumor (*p* = 0.084) were marginally correlated with OS (Table 2).

**Table 2:** Multivariate analysis of overall survival

**Variable**	**HR**	**95% confidence interval**		***p-*** **value**
		**lower**	**upper**	
T stage (T3-4 vs. T1-2)	1.368	0.863	2.168	0.182
GTV (<159 cm^3^ vs. ≥159 cm^3^)	0.564	0.314	1.014	0.056
KPS status (>70 vs. =70)	0.873	0.496	1.437	0.683
Thoracic radiation dose (<63 Gy vs. ≥63 Gy)	1.649	1.056	2.576	0.028
Response of primary tumor (SD + PD vs. CR + PR)	1.534	0.945	2.492	0.084
Chemotherapy (≥4 vs. <4 cycles)	0.804	0.507	1.274	0.353
Metastatic organ (bone with other organs vs. bone only)	1.880	1.203	2.937	0.006

## Discussion

This study sought to investigate whether combining systemic chemotherapy with radiotherapy in the treatment of the primary thoracic tumor could further improve survival in NSCLC patients with bone metastases. The results of the current study showed that radiotherapy ≥63 Gy to the primary tumor, and having only bone metastatic disease were independent prognostic factors for better OS in stage IV NSCLC patients treated with concurrent chemoradiotherapy. Radiation dose to the primary tumor ≥63 Gy remained significant when patients with bone metastases only and those with both bone and other organ metastases were analyzed separately. In accordance with a previous publication [9], our results also suggested that aggressive radiation to the primary tumor may improve survival in a subset of such NSCLC patients with bone metastases.

Lopez *et al.* reported that patients who had smaller tumor volumes had longer OS [9]. Our results showed that patients with a GTV <159 cm^3^ tended to have longer OS than those with a GTV ≥159 cm^3^. On multivariate analysis, GTV was marginally correlated with OS in this study. Higginson *et al.* reported that the status of the primary tumor was associated with OS in NSCLC patients with metastases [12]. For the subset of patients who had bone metastases only, patients with T1-2 disease had longer OS than those with T3-4. The results from these studies suggest that the status of the primary tumor should be taken under consideration; those with early T-stage and small volume tumors may obtain more benefit from aggressive radiation for their primary tumor. Radiation for the metastases was not associated with OS in the current study, probably because most of the patients (84%) did not receive radiotherapy for their metastatic disease, thus making it difficult to detect an advantage among patients who received radiotherapy for metastatic disease; and for most of the patients who received radiotherapy for their metastatic disease, it was of a palliative nature.

Hellman *et al.* proposed the notion of oligometastases to indicate the presence of limited metastases and suggested the existence of an intermediate clinical state between localized disease and widespread disease [13]. Aggressive therapy for the primary tumor and metastatic lesions in NSCLC patients with oligometastases may produce better OS [9,10,14]. Our results also showed that radiation to metastatic sites displayed a trend towards improving OS in patients with only bone oligometastases.

The recommended number of chemotherapy cycles for stage IV NSCLC is 4–6 according to the ASCO guideline [15]. We then evaluated patients with and without other organ metastases besides bone metastases separately. Univariate analysis showed that the number of chemotherapy cycles was not correlated with OS for patients who had bone metastases only. However, the subset of patients who had both bone and other organ metastases, and who had received ≥4 cycles of chemotherapy, had longer OS. Our findings suggest that the status of the metastatic disease may also be used as a criterion to decide the number of chemotherapy cycles for patients with bone metastases, when they receive radiation to the primary site.

There is a limitation of the current study in that the imaging data of some patients were not gained to evaluate patterns of failure and the relationship between OS and local control of the primary tumor. Nearly 50% of stage IV NSCLC patients experienced local recurrence in initially involved sites, and local control and status of the primary tumor has been associated with OS [12,16,17]. Several publications have confirmed that a higher radiation dose was associated with improved local tumor control and OS in patients with NSCLC [9,18]. Although the relationship between OS and local tumor control was not evaluated in this study, our results showed that the higher radiation dose to the primary tumor was correlated with OS. Thirteen of 58 patients died owing to local recurrence accompanied by distant metastases, and only six patients died of local recurrence alone. Of these six cases, four involved a radiation dose <40 Gy. These findings also suggest that the local dose used for the primary tumor played an important role in prolonging the survival of NSCLC patients with bone metastases. Because of the retrospective nature of the current study, a randomized trial is necessary to evaluate the causal effect of radiation dose on OS.

The findings from the current study can be summarized as follows. First, a higher radiation dose (≥63 Gy) to the primary tumor was significantly associated with better OS in both univariate and multivariate analysis. Second, although patients with only bone metastases had better OS than those who had both bone and other organ metastases, a higher radiation dose remained significant when patients who had only bone metastases and those who had both bone and other organ metastases were analyzed separately. Moreover, for patients who had only bone metastases, the T-stage of their primary tumor was associated with OS. In conclusion, aggressive thoracic radiation plays an important role in improving OS in NSCLC patients with bone metastases.

## Competing interests

The authors have no competing interests to declare.

## Authors’ contributions

BL designed the study, W-WO, S-FS, ZM, Y-XH, Q-SL, Y-CG and H-QL collected the data. W-WO, S-FS, ZM and BL undertook the data analyses and interpretation, and wrote the report. W-WO and BL carried out the statistical analyses. All authors read and approved the final manuscript.
